# The complete chloroplast genome sequence of *Dalbergia oliveri*

**DOI:** 10.1080/23802359.2020.1714501

**Published:** 2020-01-20

**Authors:** Jinfeng Zhang, Yunqing Li, Sokh Heng, Yi Wang

**Affiliations:** a Laboratory of Forest Plant Cultivation and Utilization, Yunnan Academy of Forestry, Kunming, People’s Republic of China;; b Institute of Forest and Wildlife Research and Development Forestry Administration, Phnom, Cambodia

**Keywords:** *Dalbergia oliveri*, chloroplast, Illumina sequencing, phylogenetic analysis

## Abstract

The first complete chloroplast genome (cpDNA) sequence of *Dalbergia oliveri* was determined from Illumina HiSeq pair-end sequencing data in this study. The cpDNA is 156,750 bp in length, contains a large single copy region (LSC) of 156,750 bp and a small single copy region (SSC) of 19,510 bp, which were separated by a pair of inverted repeats (IR) regions of 25,687 bp. The genome contains 127 genes, including 82 protein-coding genes, eight ribosomal RNA genes, and 37 transfer RNA genes. The overall GC content of the whole genome is 35.9%, and the corresponding values of the LSC, SSC, and IR regions are 33.4%, 28.8%, and 42.8%, respectively. Further phylogenomic analysis showed that *D. oliveri* and other species of Dalbergia genus clustered in a unique clade in Dalbergieae with Fabaceae family.

*Dalbergia oliveri* is the species of the genus *Dalbergia* within the family Fabaceae. The timber of *D. oliveri* is used for making mahogany furniture, handicraft carving, sports equipment (Xu et al. 2018). As a traditional Thai herbal medicine, the heartwood of *D. oliveri* is mainly used for anti-inflammatory and cancer treatment (Liu et al. 2017). Isoflavonoids isolated from *D. oliveri* is used as inhibitors (Ito et al. 2003) and to control a mosquito vector (Pluempanupat et al. 2013). Therefore, *D. oliveri* has huge value. However, there have been no genomic studies on *D. oliveri*.

Herein, we reported and characterized the complete *D. oliveri* plastid genome. The GenBank accession number is MN823694. One *D. oliveri* individual (specimen number: 201907026) was collected from Puwen, Yunnan Province of China (23°31′39″N, 101°37′21″E). The specimen is stored at Yunnan Academy of Forestry Herbarium, Kunming, China and the accession number is ZJFEP115. DNA was extracted from its fresh leaves using DNA Plantzol Reagent (Invitrogen, Carlsbad, CA, USA).

Paired-end reads were sequenced by using Illumina HiSeq system (Illumina, San Diego, CA). In total, about 23.7 million high-quality clean reads were generated with adaptors trimmed. Aligning, assembly, and annotation were conducted by CLC de novo assembler (CLC Bio, Aarhus, Denmark), BLAST, GeSeq (Tillich et al. 2017), and GENEIOUS v 11.0.5 (Biomatters Ltd, Auckland, New Zealand). To confirm the phylogenetic position of *D. oliveri*, other thirteen species of *Dalbergieae* with Fabaceae family from NCBI were aligned using MAFFT v.7 (Katoh and Standley 2013). The Auto algorithm in the MAFFT alignment software was used to align the sixteen complete genome sequences and the G-INS-i algorithm was used to align the partial complex sequences. The maximum likelihood (ML) bootstrap analysis was conducted using RAxML (Stamatakis 2006); bootstrap probability values were calculated from 1000 replicates. *Lupinus albus* (KJ468099) and *Lupinus westianus* (MG252262) were served as the out-group.

The complete *D. oliveri* plastid genome is a circular DNA molecule with the length of 156,750 bp, contains a large single copy region (LSC) of 85,866 bp and a small single copy region (SSC) of 19,510 bp, which were separated by a pair of inverted repeats (IR) regions of 25,687 bp. The overall GC content of the whole genome is 35.9%, and the corresponding values of the LSC, SSC, and IR regions are 33.4%, 28.8%, and 42.8%, respectively. The plastid genome contained 127 genes, including 82 protein-coding genes, 8 ribosomal RNA genes, and 37 transfer RNA genes. Phylogenetic analysis showed that *D. oliveri* and other species of Dalbergia genus clustered in a unique clade in *Dalbergieae* with Fabaceae family (Figure 1). The determination of the complete plastid genome sequences provided new molecular data to illuminate the Fabaceae family evolution.

**Figure 1.**
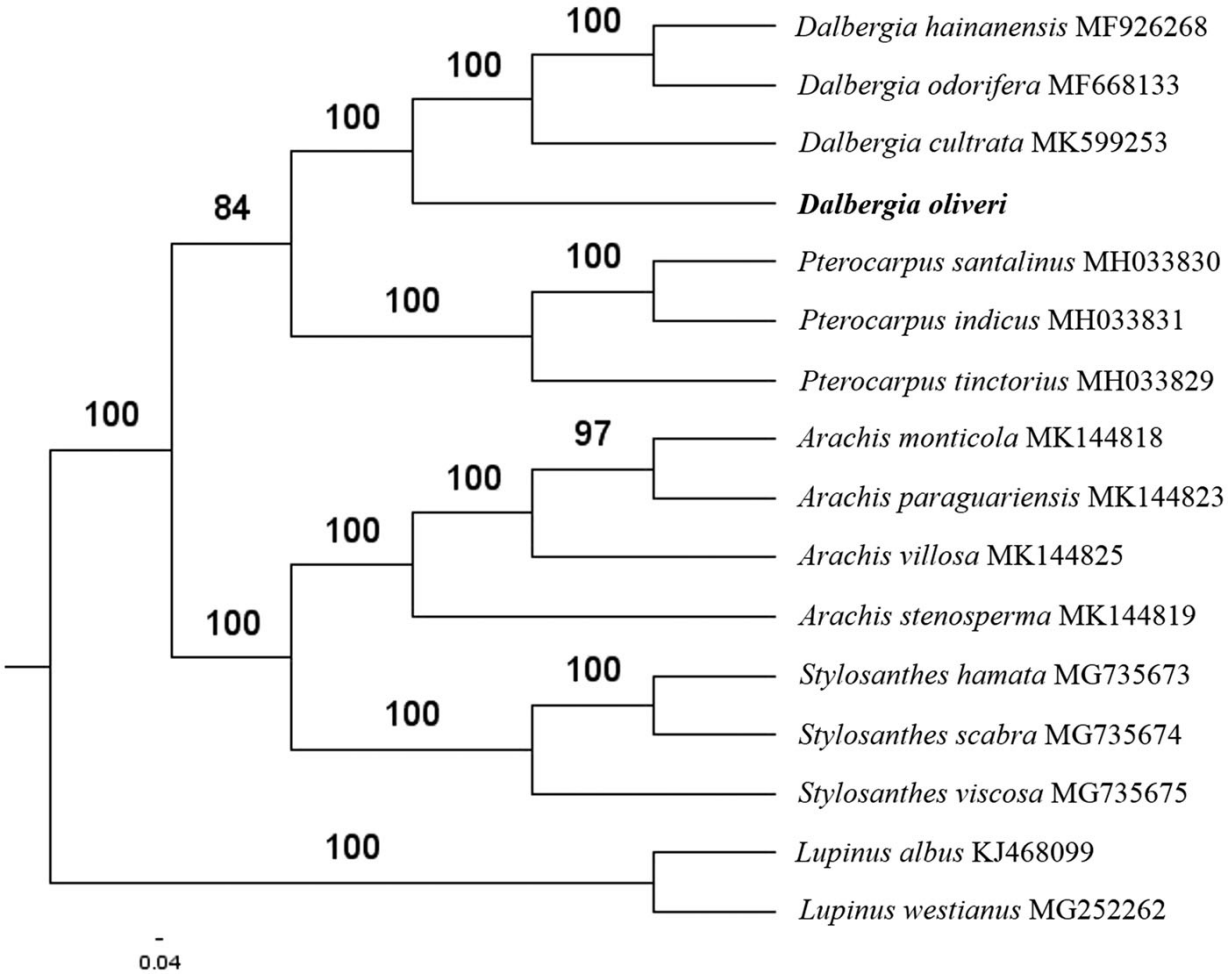
The maximum-likelihood tree based on the fourteen chloroplast genomes of *Dalbergieae* with Fabaceae family. The bootstrap value based on 1000 replicates is shown on each node.
